# Combined 22q11.1-q11.21 deletion with 15q11.2-q13.3 duplication identified by array-CGH in a 6 years old boy

**DOI:** 10.1186/1755-8166-4-6

**Published:** 2011-02-23

**Authors:** Emmanouil Manolakos, Catherine Sarri, Annalisa Vetro, Konstantinos Kefalas, Eleni Leze, Christalena Sofocleus, George Kitsos, Konstantina Merou, Haris Kokotas, Anna Papadopoulou, Achilleas Attilakos, Michael B Petersen, Sofia Kitsiou-Tzeli

**Affiliations:** 1 Bioiatriki S.A., Laboratory of Genetics, Athens, Greece; 2 Department of Genetics, Institute of Child Health, St Sophia's Children's Hospital, Athens, Greece; 3 Dipartimento di Patologia Umana ed Ereditaria, Universita di Pavia, Pavia, Italy; 4 Department of Medical Genetics, Athens University School of Medicine, St Sophia's Children's Hospital, Athens, Greece; 5 Department of Ophthalmology, University of Ioannina, Ioannina, Greece; 6 Third Department of Pediatrics, University of Athens School of Medicine, Attikon University Hospital, Athens, Greece

## Abstract

**Background:**

Deletions of chromosome 22q11 are present in over 90% of cases of DiGeorge or Velo-Cardio-Facial syndrome (DGS/VCFS). 15q11-q13 duplication is another recognized syndrome due to rearrangements of several genes, belonging to the category of imprinted genes. The phenotype of this syndrome varies but has been clearly associated with developmental delay and autistic spectrum disorders. Co-existence of the two syndromes has not been reported so far.

**Results:**

Here we report a 6-year-old boy presenting growth retardation, dysmorphic features and who exhibited learning difficulties. Fluorescence in situ hybridization (FISH) analysis of the proband revealed a deletion of DiGeorge Syndrome critical region (TUPLE). Array-CGH analysis revealed an interstitial duplication of 12 Mb in size in the area 15q11.2-q13.3, combined with a 3.2 Mb deletion at region 22q11.1-q11.21. FISH analysis in the mother showed a cryptic balanced translocation between chromosome 15 and chromosome 22 (not evident by classic karyotyping).

**Discusion:**

The clinical manifestations could be related to both syndromes and the importance of array-CGH analysis in cases of unexplained developmental delay is emphasized. The present case further demonstrates how molecular cytogenetic techniques applied in the parents were necessary for the genetic counseling of the family.

## Background

DiGeorge syndrome and Velo-Cardio-Facial syndrome (DGS/VCFS) are the result of deletions of chromosome 22q11.2 in over 90% of cases [1]. The cardinal features and symptoms are cellular immunodeficiency due to thymus hypo or aplasia, hypocalcemia because of absence of the parathyroid, congenital heart defect with high mortality and morbidity, and typical faces [2]. The prevalence of the syndrome is estimated (probably underestimated) to approximately 1:4,500 and represents one of the commonest genetic diseases [3].

The 4 Mb 15q11-q13 region, containing three biallelically expressed genes, which encode receptor subunits for the inhibitory neurotransmitter gamma-aminobutyric acid (GABRB3, GABRA5 and GABRG3), is prone to structural rearrangements [4,5]. Duplications of this region occurring on the maternal chromosome, have been associated with a complex neurobehavioral phenotype that often includes language delay, seizures and autism (OMIM# 608636) [6-11,4].

Clayton-Smith et al (1993) had initially reported a patient with a 15q11-q13 duplication including the Angelman syndrome critical region (ASCR), who had ataxia and moderate developmental delay, particularly of language, but neither epilepsy nor behavioural problems [12]. Subsequently, Bundey et al (1994) reported a boy with mental retardation, infantile autism, ataxia, and seizures, who had a more extensive interstitial duplication of 15q11-q13, including the critical regions for Prader Willi syndrome (PWS) and AS on the maternally derived chromosome [13]. Since then, various similar cases have been published and according to the literature duplications of the 15q11-q13 region constitute the most frequently reported chromosomal aberration in individuals with Autism Spectrum Disorders (ASDs) [11]. Recently, our team reported the largest contiguous de novo interstitial duplication (15q11.2-q14) of the PWS/AS region (17.7 Mb) of maternal origin [14]. The study though by Szafranski et al (2010) of a cohort of patients with small duplications (<1.6 Mb) at 15q13.2-q13.3 involving the CHRNA7 gene suggested association with developmental delay, mental retardation, muscular hypotonia and various neuropsychiatric disorders but did not prove clinical significance [15].

The use of array-CGH has increased the resolution available to studies of chromosomal abnormalities, from ≥ 5 Mb, achieved by metaphase Fluorescence in situ hybridization (FISH), to >100 kb, or the size of a Bacterial Artefact Clone (BAC clone), to <16.4 kb average spatial resolution with oligonucleotide arrays. Especially the higher resolution arrays are able to define the breakpoint of rearrangements and to accurately detect copy number.

Here we present the clinical and molecular findings in a 6-year-old boy with a duplication of about 12.9 Mb that involves the region 15q11.2-q13.3 along with a deletion of approximately 3.2 Mb on the long arm of chromosome 22, at the region 22q11.1-q11.21. The sizes and breakpoints of the deletion and duplication were determined through oligo array-CGH analysis. To our knowledge this is the first case of concurrence of DiGeorge syndrome with a maternally inherited 15q12.2-q13.3 duplication. The aim of this study is to correlate the molecular findings with the proband's clinical phenotype and to demonstrate that molecular cytogenetic techniques used in the parents were necessary for the genetic counseling of the family.

## Case presentation

The proband, a 6-year-old boy, is the first child of non-consanguineous, healthy 30 year-old parents. The family history was unremarkable. Pregnancy was uneventful, there was no prenatal exposure to teratogens but the prenatal ultrasound examination showed exadactyly (right hand). Amniocentesis revealed a normal karyotype 46,XY. The proband was born at 35 weeks of gestation by normal delivery, with birth weight of 2600 g, length 49 cm and head circumference 31 cm, all within normal range for the age of gestation. Apgar scores were 9/1" and 10/5" and he had an uncomplicated perinatal period. At birth he presented radial exadactyly and syndactyly of 3^rd ^and 4^th ^fingers (right) as well as a radial ulnar (left) rudimentary supernumerary finger and other malformations. Ultrasound of brain/abdomen and cardiovascular examination were normal.

Radiological re-examination of the right hand at the first year of life showed syndactyly of the 3^rd ^and 4^th ^fingers and a supernumerary dysplastic finger which was surgically removed. Two years later surgical axonal repair of the syndactyly was performed and since then the patient has been regularly followed up by orthopaedists.

His initial motor development was slightly retarded; head control was achieved at the age of 5 months, unsupported sitting at 16 months and walking at 18 months. His language acquisition was also delayed. Early developmental intervention with physiotherapy and sessions for speech improvement showed gradual but not remarkable progress. Audiologic evaluation revealed congenital sensorineural hearing loss bilaterally (80-90 dB) which was not attributed to the 35delG mutation of the connexin 26 gene (*GJB2*) after molecular analysis by PCR [16]. Fragile-X syndrome was also excluded, while MRI of the brain was within normal limits.

During three hospitalizations at the age of 4 and 5 years due to recurrent right lung pneumonia, extended immunologic studies excluded immunodeficiency. Routine blood and urine biochemical investigations as well as thorough metabolic and endocrinological evaluation showed no abnormality while ophthalmologic evaluation revealed hypermetropy. The examination of internal organs by ultrasonography was also negative for any pathology.

The boy was referred for genetic evaluation at the age of 6 years. He showed growth retardation [height at 105 cm (<3^rd ^centile), weight 17 kg (3^rd ^centile), head circumference 49.5 cm (3^rd ^centile)], made 2-5 word phrases and exhibited learning difficulties. Dysmorphic features included facial weakness, blepharoptosis (not myopathetic type), downslanting palpebral fissures, epicanthus, full cheeks, thick/small/low-set/dysplastic ears, high-arched palate, chin dimpling, bulbous nasal tip, and microstomia.

## Results

**Figure 1:**
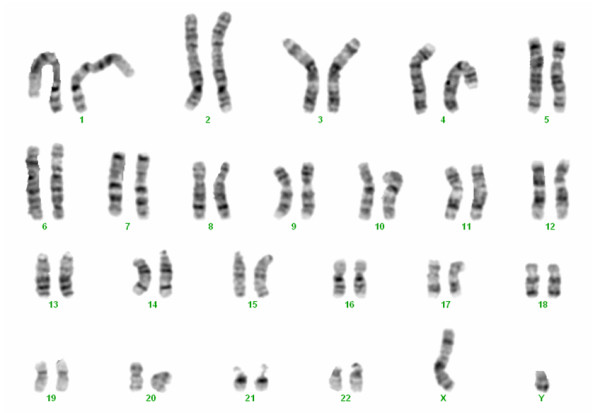
**Patient's karyotype (GTG-banding)**.

Chromosome analysis of the proband was performed using GTG-banding techniques on stimulated blood lymphocytes. Cytogenetics revealed a normal karyotype 46,XY (Figure 1).

**Figure 2:**
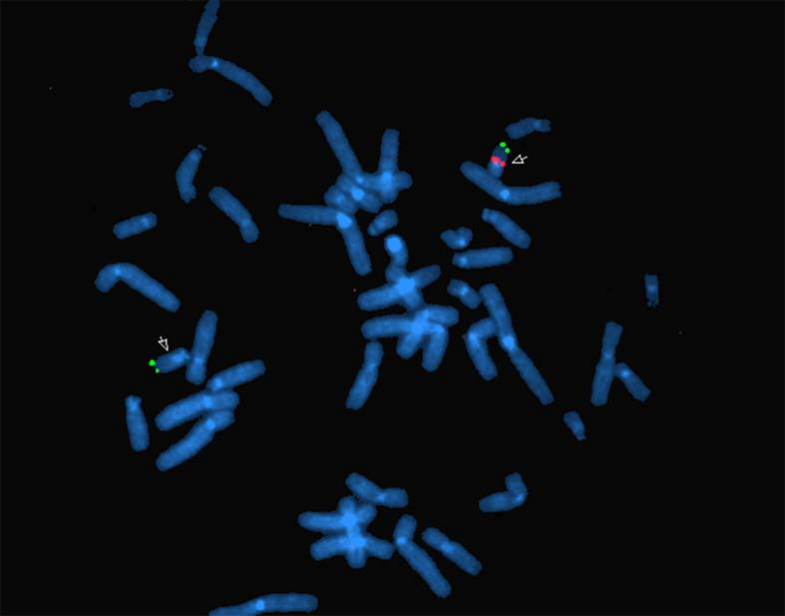
**FISH analysis of the proband using a probe (labeled red) specific for DiGeorge Syndrome critical region (TUPLE)**. A single red signal (white arrow) can be seen on the normal chromosome 22, while the red signal is absent on the chromosome 22 with the deletion. Two green signals are seen on the subtelomeres of both homologues (control probe).

Due to the clinical findings fluorescence *in situ *hybridization (FISH) using a probe specific for DiGeorge Syndrome critical region (TUPLE) was performed. A single red signal could be seen on the normal chromosome 22 (Figure 2), while the red signal was absent on the homologous chromosome 22. Two green signals were seen on the subtelomere of both homologues (control probe).

**Figure 3:**
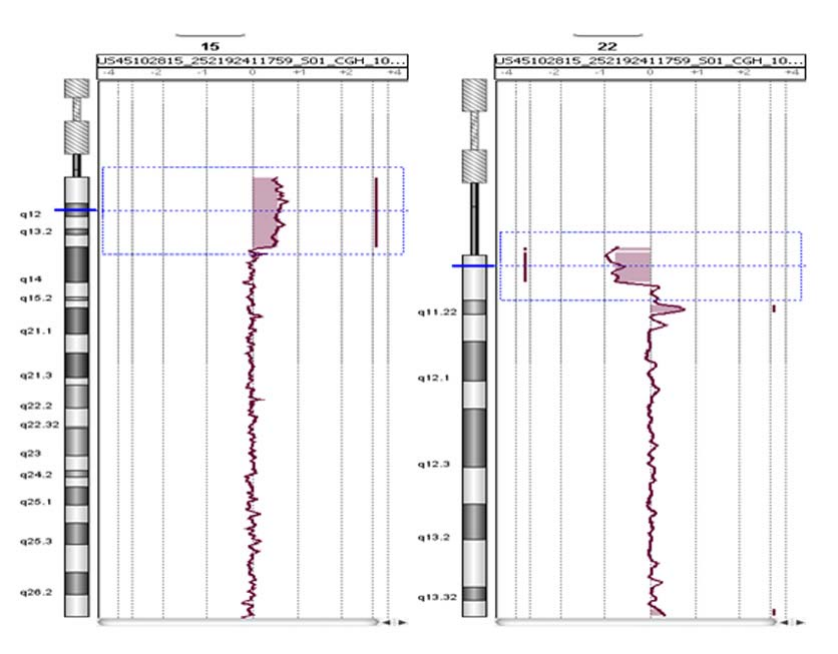
**Molecular characterization of the proband showed a 3.2 Mb 22q11.1q11.21 deletion and a 12.9 Mb 15q11.2-q13.3 duplication**. The small duplicated region visible under the 3.2 Mb deletion on chromosome 22q is a known benign CNV related to the reference DNA used in the experiment (NA10851).

Due to atypical clinical findings molecular karyotyping was performed through array-CGH analysis on the proband's DNA by using an Agilent 60 K array platform. This analysis showed a 22q11.1-q11.21 deletion of about 3.2 Mb, with distal breakpoint falling between 18,691,704 bp (last deleted oligomer) and 19,084,422 bp (first normal oligomer). The first oligomer on the platform for proximal 22q maps at 15,476,855 bp and resulted to be deleted. The analysis also detected a duplication of about 12.9 Mb that involves the 15q11.2-q13.3 region, with distal breakpoint falling between 31,288,255 bp (last duplicated oligomer) and 31,339,610 bp (first normal oligomer). The first oligomer on the platform for proximal 15q maps at 18,362,555 bp and was duplicated (Figure 3).

**Figure 4:**
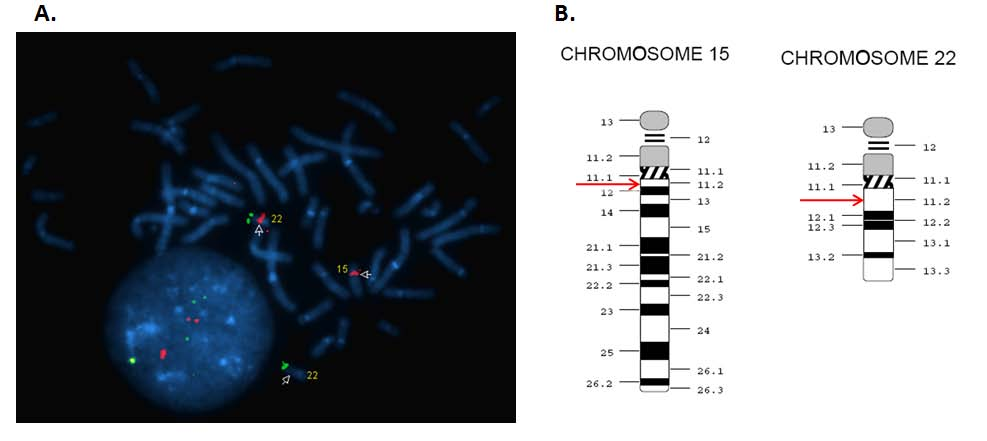
**FISH analysis of the mother using a probe (labeled red) specific for DiGeorge Syndrome critical region (TUPLE) and schematic representation of the corresponding breakpoints on chromosomes 15 and 22**. A. A red signal together with a green signal (subtelomere specific) can be seen on the normal chromosome 22. However in the homologous chromosome 22 only one green signal can be seen, while the red signal is translocated on to a chromosome of the D group depicting that the mother bears a cryptic translocation. B. An ideogram scheme of the breakpoints (red arrows) on chromosomes 15 and 22 that resulted in a balanced translocation in the mother.

Parental karyotypes were normal. Parental FISH analysis was requested and the mother was found to carry a cryptic balanced translocation between chromosome 15 and chromosome 22 with a 46,XX,t(15;22)(q11.2;q11.2) karyotype (Figure 4) using the following two probes [TUPLE and TBX1 DiGeorge Region probes (Cytocell)]. The karyotype of the proband was defined as follows: 46,XY,ish der(22)t(15;22)(q11.2;q11.2)(TUPLE-,TBX-)mat.

According to fragment analysis three alleles were observed in the proband using microsatellite markers D15S122, D15S113 and D15S97, all within the PWS/AS critical region. The markers were not informative of the parental origin of the partial trisomy 15. Biparental inheritance of chromosome 15 was observed with marker 85CA.

## Discussion

The long arm of chromosome 15 and essentially the 15q11.2-q14 PWS/AS region is highly susceptible to clinically important genomic rearrangements, including interstitial deletions, duplications, and triplications [17,18]. Transcriptional regulation of the 15q11-q14 region is highly complex, involving multiple allele-specific epigenetic marks, including DNA methylation, histone modification patterns and DNAse hypersensitive sites [19]. The area also contains a cluster of snoRNA and noncoding RNAs, which are under specific imprinting regulation [20]. Repeat sequences, such as low copy repeats (LCR) are thought to mediate misalignment of the region during meiosis, leading to unequal recombination events [14].

Duplications reported so far usually share two proximal Breakage Points (BPs) (BP1 and BP2) and usually one distal BP3 and include *PWACR*. More distal BPs (BP4 and BP5) are involved in large inv-dup(15)s and intrachromosomal triplications [17,21]. The frequency of 15q11-q13 interstitial duplications is estimated at 1:600 individuals referred with developmental delay [22].

Interstitial duplications of proximal 15q that do not include the PWS/AS critical region have *no *clinical effect, are usually familial, and may be considered *normal variants *[23]. Patients with interstitial duplications of the PWS/AS locus have an abnormal phenotype that includes developmental delay, particularly affecting speech and language; varying degrees of mental retardation; autism or autistic features; motor coordination difficulties; and mild or no dysmorphic features [24]. The phenotype is highly variable, even among members of the same family carrying identical rearrangements [24] and can in some cases manifest as developmental language disorder and dyspraxia, without autism [25]. Increased dosage of the PWS/AS critical gene region between BP2 and BP3 positively correlates with phenotypic severity in patients with 15q11-q13 duplications, however clinical heterogeneity in patients is not explained by variation in breakpoints only, suggesting that additional factors contribute to clinical complexity. At least 33 cases of interstitial duplications of the 15q11-q13 region have been reported in association with ASDs, according to the Autism Chromosome Rearrangement Database (http://projects.tcag.ca/autism/).

Overexpression of maternally imprinted genes through duplication of chromosome 15q11-q14 also displays parent-of-origin effects, with maternal duplications being associated with a complex neurobehavioral phenotype that often includes autism, cognitive deficits and seizures [10,11,4]. Twenty-five cases with maternal origin of the intrachromosomal duplication showed a range of phenotypic features from severe mental retardation, developmental delay, seizures, nonspecific hypotonia, and mild facial anomalies to only developmental delay and autism [16,24,26,27].

One case of autistic disorder associated with a paternally derived unbalanced translocation leading to duplication of chromosome 15pter-q13.2 has been reported, suggesting that biallelically expressed genes on proximal 15q contribute to the autism phenotype [28].

Regarding the developmental outcome of our proband, he lacked seizures and autistic behavior which have been associated with 15q11-q13 duplication. He had marked psychomotor delay, expressed primarily as poor speech, probably attributed to the 15q11-q13 duplication since 22q11 deletion mostly causes mild retardation [1,14].

Congenital heart defects, mainly of the cardiac outflow tract and aortic arch, commonly severe, are present in 75% of patients with DiGeoge syndrome and are possibly associated with *TBX1 *haploinsufficiency [29]. In our proband though, the congenital heart defect was restricted to transient patent foramen ovale which is a simple and non life-threatening anomaly. Within the spectrum of DGS/VCFS, small ears and deafness are listed, but usually the latter is conductive and when sensorineural, as in our patient, is bilateral. Our proband was lacking several of the most common features referred within the clinical spectrum of del22q11.2-q11.21, as immunodeficiency, various endocrinological manifestations and hypocalcemia [3]. The polydactyly (either pre- or postaxial) has also been described, but not syndactyly that the patient also demonstrated. The frequency of inherited 22q11.2 deletions reported by Ryan et al 1997 [30], is much higher than in previous studies (28% versus 10%, respectively) and it has been suggested that both parents should be investigated in any case of DGS/VCFS [31,32]. Mosaic cases of 22q11.2 microdeletion syndrome seem very rare [33].

Another case of a cryptic translocation in one of the healthy parents was reported [34] in a case where FISH showed a t(1;17)(q44;p13.2) translocation in the father, which subsequently enabled the characterization of the der(1) chromosomal abnormality in the index patient. It is important that cryptic translocations are kept in mind, and that parental karyotypes are analyzed in cases of mentally retarded children with normal karyotype.

Our report adds another case of maternal origin of 15q11-q13 duplication in the current literature and shows again the syndrome variability which becomes more obscure when accompanied by another chromosomal abnormality, as 22q11 deletion. Furthermore, it represents, as far as we know, the first case of concurrence of 22q11.1-q11.21 deletion (mat) with 15q11.2-q13.3 duplication. It illustrates the value of thorough clinical evaluation and multiple genetic tests in patients with unexplained developmental delay and major congenital anomalies as well as in their parents with the scope of excluding possible balanced translocations predisposing to genetic risk. We suggest that reporting of similar well-characterized clinical cases with clearly delineated breakpoints of the duplicated region will clarify the contribution of specific genes to the phenotype and will offer accurate genotype versus phenotype correlation that should allow a more precise prognosis.

## Materials and methods

### Chromosome analysis and FISH

Chromosome analysis was carried out on PHA-stimulated lymphocytes with standard GTG-banding technique. FISH studies were performed by use of the standard method described by Pinkel et al (1988) [35]. Hybridization signals were detected by two digoxigenin (DIG) - probes according to the manufacturer's instructions. The probes used were DiGeorge and 22q13.3 Deletion Syndrome Probe Combinations (Cytocell). Two out of three commercially available probes from Cytocell were used:

1) VCFS TUPLE 1 Region deletion probe, labeled in red which measures approximately 120 kb and covers the entire TUPLE1 gene and flanking DNA,

2) TBX1 Region deletion probe labeled in red, which is approximately 213 kb, and contains the D22S1627 marker. Both are DIG-probes directly labeled with a red fluorochrome (Texas Red spectrum) and accompanied by the 22 telomere control probe directly labeled with a green fluorophore (FITC spectrum).

A total of 40 metaphases from each probe of the proband and a total of 20 metaphases from each probe of the carrier mother were examined after the hybridization and the number and relative position of signals were recorded. Signal distribution and intensity from the normal chromosome 22 homologue acted as an internal control for the efficiency and stringency of the hybridization experiment. Analysis was performed by use of a conventional Zeiss epifluorescent Axioskop 2plus microscope, and images were captured, enhanced and analyzed by use of Cytovision (Applied Imaging) software. Both the conventional cytogenetics and FISH results were described according to the 2009 International System for Human Cytogenetics Nomenclature (ISCN) [36].

### Molecular karyotyping

Molecular karyotyping was carried out on DNA extracted from whole blood of the patient and both his parents according to standard procedures. All the experiments were conducted through oligo array-CGH platforms (SurePrint G3 Human CGH Microarray, 8 × 60 K, Agilent Technologies, Santa Clara, CA, USA). Briefly 500 ng of the proband and of a sex-matched reference DNAs (NA10851, Coriell Cell Repositories) were processed according to the manufacturer's protocol. Fluorescence was scanned in a dual-laser scanner (DNA Microarray Scanner with Sure Scan High-Resolution Technology, Model G2565CA, Agilent Technologies, Santa Clara, CA, USA) and the images were extracted and analyzed through Agilent Feature Extraction Software (v10.5.1.1). Graphical overview was obtained using the DNA Analytics software (v4.0.73). Changes in test DNA copy number at a specific locus were observed as the deviation of the log_2 _ratio value of 0 of at least three consecutive probes. The quality of each experiment was assessed by using a parameter provided by Agilent software (QC metric). Copy number changes identified in the samples were visualized by using the UCSC Genome Browser website (http://genome.ucsc.edu) and also compared to the Database of Genomic Variants (http://projects.tcag.ca/variation) to exclude copy number changes considered as benign variants. The positions of oligomers refer to the Human Genome March 2006 (versions NCBI 36, hg18) assembly. The DECIPHER (https://decipher.sanger.ac.uk/) and ECARUCA (http://agserver01.azn.nl:8080/ecaruca/ecaruca.jsp) databases were expedient as resources to aid genotype-phenotype correlation.

### Fragment analysis

Chromosome 15 microsatellite markers 85CA, D15S122, GABRB3, D15S113, CYP19, D15S97, and FES were genotyped. The DNA fragments were amplified by polymerase chain reaction (PCR) and the products were further diluted in Hi-Di™ formamide and GeneScan™ 500 LIZ^® ^which were used as an internal lane size standard in order to enable automated data analysis and precise DNA fragment size comparisons between electrophoresis runs. The final products were separated by capillary electrophoresis on an ABI 310 genetic analyzer (Applied Biosystems, Foster City, CA, USA) according to the manufacturer's protocol and the results were evaluated using the GeneMapper v4.0 software (Applied Biosystems, Foster City, CA, USA).

## Consent

Written informed consent was obtained from the parents of the patient for publication of this case report and accompanying images. A copy of the written consent is available for review by the Editor-in-Chief of this journal.

## Competing interests

The authors declare that they have no competing interests.

## Authors' contributions

EM wrote the manuscript; SKT, EL and AA coordinated the clinical analysis of the patient; EM, CS and KM performed the cytogenetic analysis; AV and KK signed out the molecular cytogenetic results; HK, CS and AP were responsible for the molecular analysis; GK performed the ophthalmologic examination; MBP and SKT coordinated the study. All authors have read and approved the manuscript.
