# Combined 22q11.1-q11.21 deletion with 15q11.2-q13.3 duplication identified by array-CGH in a 6 years old boy

**DOI:** 10.1186/1755-8166-4-11

**Published:** 2011-04-16

**Authors:** Emmanouil Manolakos, Catherine Sarri, Annalisa Vetro, Konstantinos Kefalas, Eleni Leze, Christalena Sofocleus, George Kitsos, Konstantina Merou, Haris Kokotas, Anna Papadopoulou, Achilleas Attilakos, Michael B Petersen, Sofia Kitsiou-Tzeli

**Affiliations:** 1 Bioiatriki S.A., Laboratory of Genetics, Athens, Greece; 2 Department of Genetics, Institute of Child Health, St Sophia's Children's Hospital, Athens, Greece; 3 Dipartimento di Patologia Umana ed Ereditaria, Universita di Pavia, Pavia, Italy; 4 Department of Medical Genetics, Athens University School of Medicine, St Sophia's Children's Hospital, Athens, Greece; 5 Department of Ophthalmology, University of Ioannina, Ioannina, Greece; 6 Third Department of Pediatrics, University of Athens School of Medicine, Attikon University Hospital, Athens, Greece

## Correction

The results section of the published article [1] state that the karyotype of the proband was defined as follows: 46,XY,ish der(22)t(15;22)(q11.2;q11.2)(TUPLE-,TBX-)mat.

The above definition contains an error and should instead be written as follows: 46,XY.ish der(15)(15pter-&gt;15q11.2::22q11.2-&gt;22qter)t(15;22)(q11.2;q11.2)mat(TUPLE-,TBX1-).
